# Development of a novel rat long-bone nonunion model and efficacy evaluation of a prostaglandin EP4 selective agonist (AKDS001) combined with iliac bone grafting

**DOI:** 10.1302/2046-3758.143.BJR-2024-0220.R1

**Published:** 2025-03-03

**Authors:** Daisuke Tateiwa, Masahiro Nishida, Joe Kodama, Hiromasa Hirai, Shinichi Nakagawa, Yuichiro Ukon, Kazuhiro Takeyama, Natsumi Yamamori, Kyoko Hirano, Masato Ikuta, Takayuki Kitahara, Takuya Furuichi, Masayuki Bun, Seiji Okada, Takashi Kaito

**Affiliations:** 1 Department of Orthopaedic Surgery, Osaka International Medical and Science Center, Osaka, Japan; 2 Laboratory for Pharmacology, Pharmaceuticals Research Center, Asahi Kasei Pharma Corporation, Shizuoka, Japan; 3 Department of Orthopaedics, University of Maryland School of Medicine, Baltimore, Maryland, USA; 4 Department of Orthopaedic Surgery, Osaka University Graduate School of Medicine, Suita, Japan; 5 Department of Orthopaedic Surgery, Osaka Rosai Hospital, Sakai, Japan

**Keywords:** Nonunion, Prostaglandin EP4 selective agonist, Bone morphogenetic protein, Iliac bone grafting, Prostaglandin, bone nonunion, agonists, rat model, bone grafting, pseudoarthrosis, fracture sites, Kirschner wire (K-wire), μCT images, bone morphogenetic protein-2

## Abstract

**Aims:**

Nonunion occurs when a fracture fails to heal permanently, often necessitating surgical intervention to stimulate the bone healing response. Current animal models of long-bone nonunion do not adequately replicate human pathological conditions. This study was intended as a preliminary investigation of a novel rat nonunion model using a two-stage surgical intervention, and to evaluate the efficacy of a selective prostaglandin E2 receptor 4 agonist (AKDS001) as a novel nonunion therapeutic agent compared with existing treatments.

**Methods:**

Initially, Sprague-Dawley rats underwent intramedullary Kirschner wire (K-wire) fixation of a femoral fracture with the interposition of a 2 mm-thick silicon disc. After three weeks, the silicon disc was removed, and the intramedullary K-wire was replaced with plate fixation while maintaining the 2 mm defect. Contrary to the control group (1) that received no treatment, the following therapeutic interventions were performed at injury sites after freshening: (2) freshening group: no grafting; (3) iliac bone (IB) group: IB grafting; (4) AKDS group: AKDS001-loaded microspheres (MS) combined with IB (0.75 mg/ml); and (5) bone morphogenetic protein (BMP) group: grafting of a BMP-2-loaded collagen sponge (10 μg; 0.10 mg/ml). After six weeks, micro-CT (μCT) and histological analysis was performed.

**Results:**

In the control group, the radiological union rate was 0%, and histological findings showed that fracture sites comprised fibrous scar tissue, resembling the histology of human nonunion. The union rates in the freshening, IB, AKDS, and BMP groups were 16.7%, 0%, 62.5%, and 50.0%, respectively. The AKDS group demonstrated a significantly higher union rate than the IB group (p = 0.026). μCT and histological analysis indicated that the quality of newly formed bone was superior in the AKDS group than in the BMP group.

**Conclusion:**

We developed a novel long-bone nonunion model. The co-therapy of AKDS001-MS and IB grafting presents a promising new treatment for nonunion.

Cite this article: *Bone Joint Res* 2025;14(3):166–175.

## Article focus

- We developed a novel long-bone nonunion model using a unique two-stage surgical technique.
- This model demonstrated that a co-therapy of selective prostaglandin E2 receptor 4 agonist (AKDS001)-loaded microspheres (MS) and iliac bone (IB) grafting can be a new effective treatment for bone nonunion.

## Key messages

- Co-therapy with AKDS001-MS and IB grafting significantly enhanced the union rate compared with IB grafting alone, demonstrating bone regeneration with high-quality bone formation.
- We developed a novel long-bone (femur) nonunion rat model that reflects the pathophysiology of human nonunion.

## Strengths and limitations

- This nonunion model reflects the chronic state of nonunion in humans without requiring a critical-sized bone defect.
- Since atrophic nonunion generally does not form callus tissue, this model is not strictly representative of clinically significant atrophic nonunion.

## Introduction

Delayed union or nonunion occurs in approximately 5% to 10% of long-bone fractures.^1^ These conditions often result in pain, stiffness, and disability in the surrounding joints, leading to work incapacity and imposing a substantial burden on patients and society.^2^ Autologous bone grafting is the gold standard for the second surgical intervention for nonunion;^3,4^ however, failure rates range from 14.9% to as high as 44.7%.^5-9^ Consequently, various alternative treatments have been explored, including the use of bone morphogenetic proteins (BMPs),^8^ low-intensity ultrasound stimulation,^10^ shockwave therapy,^11^ and cell-based therapy.^12^ However, clinical trials of these treatments have not demonstrated the expected efficacies observed in animal preclinical experiments, highlighting the need for an appropriate animal model that mimics the pathological conditions observed in humans.^13^

Previously reported animal models of nonunion, developed through critical-sized bone defects or inhibition of bone formation by periosteum (periosteum cauterization or resection),^14,15^ have shown high reproducibility but do not fully reflect the pathophysiology of human nonunion.^14^ Nonunion is a chronic condition where adequate bone healing does not occur within six to nine months after therapeutic intervention. Therefore, animal models that mimic this chronic condition, including impaired fracture healing capacity, are needed.

BMPs are potent osteoinductive growth factors clinically used to treat nonunion.^8^ While BMPs can induce new bone formation even without grafting, the quality of bone formed is lower than that achieved with autologous bone grafting.^16^ Furthermore, BMPs are associated with dose-dependent adverse events, including soft-tissue swelling, local inflammation, osteolysis, ectopic bone formation, retrograde ejaculation, and radiculitis.^16^ Prostaglandin E2 receptor 4 (EP4) agonists, which have potent osteogenic effects,^17^ have emerged as potential alternatives to BMPs. Selective EP4 agonists have shown efficacy in treating osteoporosis and enhancing fracture healing;^17^ however, their efficacy in long-bone nonunion has not yet been evaluated. Recently, we reported that a novel EP4 agonist (AKDS001) enhanced new bone formation using a human bone xenograft model, inducing new bone formation in grafted bone through minimodelling rather than remodelling.^18^

In this study, we developed a novel long-bone (femur) nonunion rat model that reflects the pathophysiology of human nonunion, and investigated the efficacy of combined AKDS001 and iliac bone (IB) grafting treatment in comparison with existing treatments.

## Methods

All animal experiments were approved by the institutional animal experimental committee (approval number 02-077-004), conducted in accordance with the National Institutes of Health Guide for the Care and Use of Laboratory Animals.^19^ We have included an ARRIVE checklist to show that we have conformed to the guidelines.

A total of 36 male eight-week-old Sprague-Dawley (SD) rats were used, based on preliminary experiments. The rats were anaesthetized with a combination of 0.15 mg/kg medetomidine (Nippon Zenyaku Kogyo, Japan) and 2.0 mg/kg midazolam (Astellas Pharma, Japan). For postoperative analgesia, 2.5 mg/kg butorphanol (Meiji Seika Pharma, Japan) was administered subcutaneously. After the first and second surgeries (detailed below), the rats were housed individually, allowed unrestricted weightbearing, and provided with food and water ad libitum. Their condition was monitored daily. The rats were euthanized under anaesthesia six weeks after the second surgery.

### Two-stage surgeries for a novel nonunion animal model

**Fig. 1:**
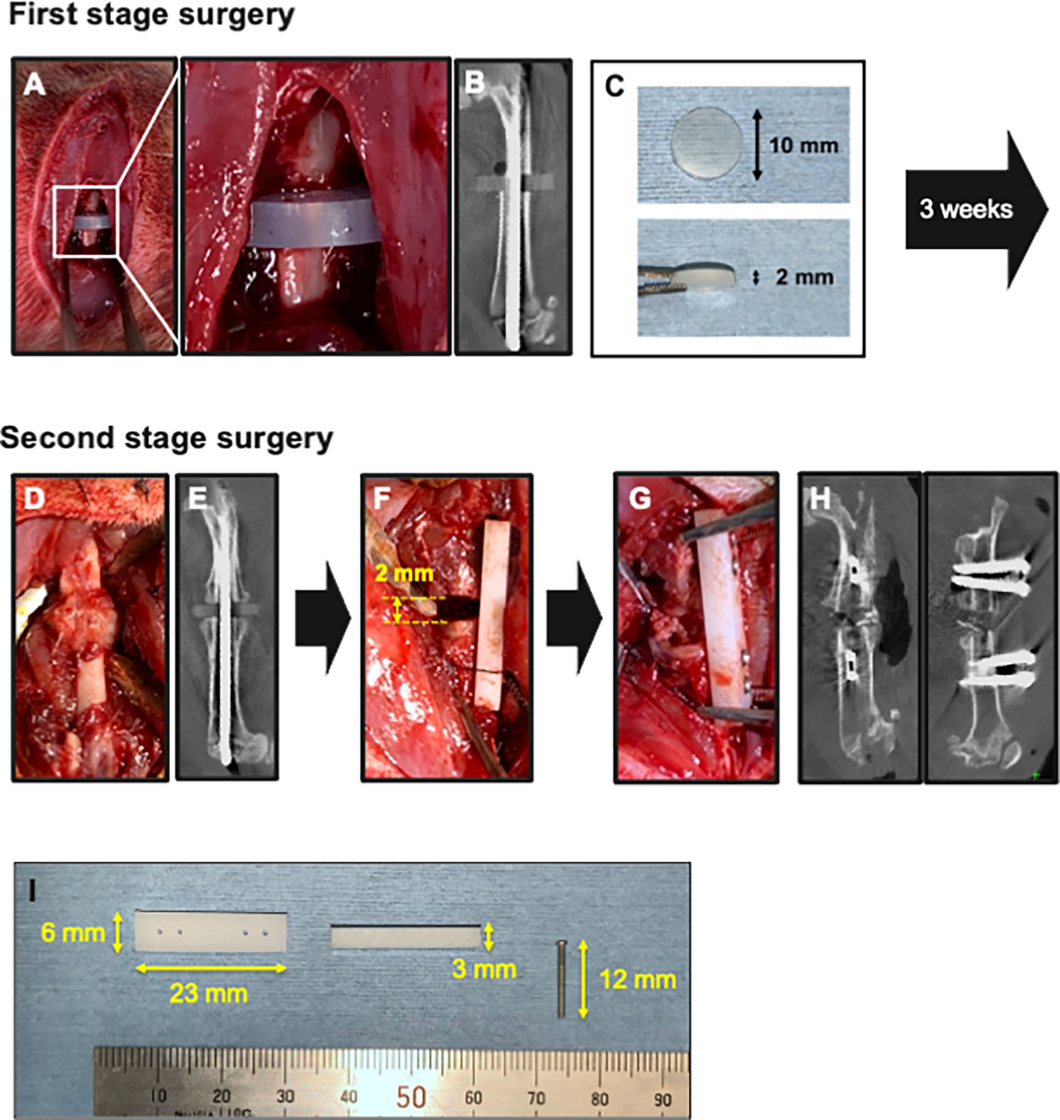
Two-stage surgical procedures of a novel nonunion model. a) First-stage surgery. An intramedullary Kirschner wire was inserted with a silicon disc interposed at the mid-diaphysis fracture site. b) In vivo micro-CT (μCT) image of the femur immediately after the first-stage surgery. c) A silicon disc (10 mm in diameter, 2 mm thick). d) and e) Second-stage surgery. Macroscopic appearance and in vivo μCT image of the femur three weeks after the first-stage surgery. Scar tissue and callus were observed around the fracture site. f) Conversion from intramedullary fixation to plate fixation, maintaining the 2 mm defect (equivalent to the thickness of the silicon disc). g) Iliac bone grafting into the gap. h) In vivo μCT images immediately after the second-stage surgery. i) A polyoxymethylene plate (23 mm long × 6 mm wide × 3 mm high) and a stainless-steel screw (φ1.2 × 12 mm).

**First-stage surgery (preparatory surgery of the model):** The right femur of each animal was exposed using a lateral approach, followed by the creation of a transverse fracture at the mid-diaphysis with a bone saw. Subsequently, the knee joint was exposed via a medial parapatellar approach, and an intramedullary Kirschner wire (K-wire; diameter 1.2 mm) was inserted retrogradely, with a silicon disc (diameter 10 mm; thickness 2 mm) interposed at the mid-diaphysis fracture site (Figures 1a and 1b). The silicon disc was created by punching out a 2 mm thick silicon rubber sheet (AS ONE Corp., Japan) (Figure 1c). To prevent dislocation, the distal end of the K-wire was bent. Finally, the muscle and skin were sutured.

**Second-stage surgery:** The second surgery was performed three weeks after the initial procedure. The right femur was re-exposed using a lateral approach. The inability to control rotational motion with the intramedullary K-wire led to callus and scar tissue formation around the fracture site (Figures 1d and 1e). To ensure mechanical stability at the fracture site, the K-wire and silicon disc were removed, and internal fixation was performed using a polyoxymethylene internal plate (23 mm long × 6 mm wide × 3 mm high; Matec, Japan) and four stainless-steel screws (φ1.2 × 14 mm; Matsumoto Industry, Japan), maintaining the 2 mm defect (silicon disc thickness) (Figures 1f and 1i). The fracture ends were freshened using a high-speed burr to remove fibrous soft-tissue, except in the no treatment (control) group.

### Osteogenic agents

AKDS001 was supplied by Asahi Kasei Pharma Corporation (Japan) and incorporated into biodegradable poly lactic-co-glycolic acid microspheres (MS), at a feed molar ratio of 1:1. During implantation, AKDS001-loaded MS were mixed with 80 mg of IB at a concentration of 0.75 mg/ml. The sustained release of AKDS001 from the MS was demonstrated for at least three weeks after implantation.^18^

An 8.0 mm diameter disc-shaped collagen sponge (CS) was prepared by punching out an absorbable CS (CollaTape; Zimmer Dental, USA). Each CS was loaded with 100 μl of phosphate-buffered saline containing 10 μg of recombinant human BMP-2 (rhBMP-2) (Z02913; GenScript, USA) at a concentration of 0.10 mg/ml, and then freeze-dried using a benchtop freeze dryer (FreeZone 2.5; Labconco, USA).

### Experimental design and graft materials

The animals were divided into five groups (Table I) as follows: 1) control group (n = 4): no grafting or freshening (only removal of the silicon disc); 2) freshening only group (n = 6): no grafting after freshening of the fracture ends; 3) IB group (n = 8): after freshening ends, grafting of 80 mg of IB (including both cortical and cancellous bone) obtained immediately before implantation from a donor SD rat (male, eight weeks old) (Figures 1g and 1h); 4) AKDS group (n = 8): grafting of AKDS001 (0.75 mg/ml)-loaded MS combined with 80 mg of IB after freshening the ends; and 5) BMP group (n = 6): grafting of rhBMP-2 (10 μg; 0.10 mg/ml)-loaded CS after freshening the fracture ends.

**Table I.** Experimental design.

Group	Grafting material
**Without freshening of fracture ends**	
(1) Control	None (only removal of silicon disc)
**With freshening of fracture ends**	
(2) Freshening only	None
(3) IB	IB (80 mg)
(4) AKDS	IB (80 mg) with AKDS001 (0.75 mg/ml)-loaded MS
(5) BMP	CS containing rhBMP-2 (10 μg; 0.10 mg/ml)

BMP, bone morphogenetic protein; CS, collagen sponge; IB, iliac bone; MS, microspheres; rhBMP-2, recombinant human BMP-2.

To investigate the fracture sites in this nonunion model, four rats were euthanized three weeks after the first operation for micro-CT (μCT) and histological analyses.

### Micro-CT analysis

The rat femora were scanned using high-resolution µCT (SkyScan 1272; Bruker, USA) with a source voltage of 80 kV and a current of 125 μA. Bony union was determined by assessing the continuity of the proximal and distal fracture ends. In successfully fused samples, bone volume (BV), tissue volume (TV), BV/TV ratio, and bone mineral density (BMD) were measured using CT-Analyser software for the Bruker µCT scanner (Blue Scientific, UK).

### Histological analysis

Tissue samples were fixed in formalin, decalcified in 10% ethylenediaminetetraacetic acid, dehydrated using ethanol, embedded in paraffin wax, and serially sectioned at 3 μm thickness. Haematoxylin and eosin and Safranin-O/Fast Green staining were performed following standard protocols. Masson-Goldner trichrome staining was conducted using the Masson-Goldner staining kit (Sigma-Aldrich, USA), and tartrate-resistant acid phosphatase (TRAP) staining was performed using the TRAP staining kit (Cosmo Bio, Japan).

### Statistical analysis

Fisher’s exact test was performed to compare bone union rates, and independent-samples *t*-test was performed to compare bone morphometric analysis results. Data were analyzed using GraphPad Prism 8.0 (GraphPad Software, USA) and are expressed as mean (SD). Statistical significance was set at a p-value of < 0.05. No animals were excluded from the analyses.

## Results

### Fracture site three weeks after the first surgery

**Fig. 2:**
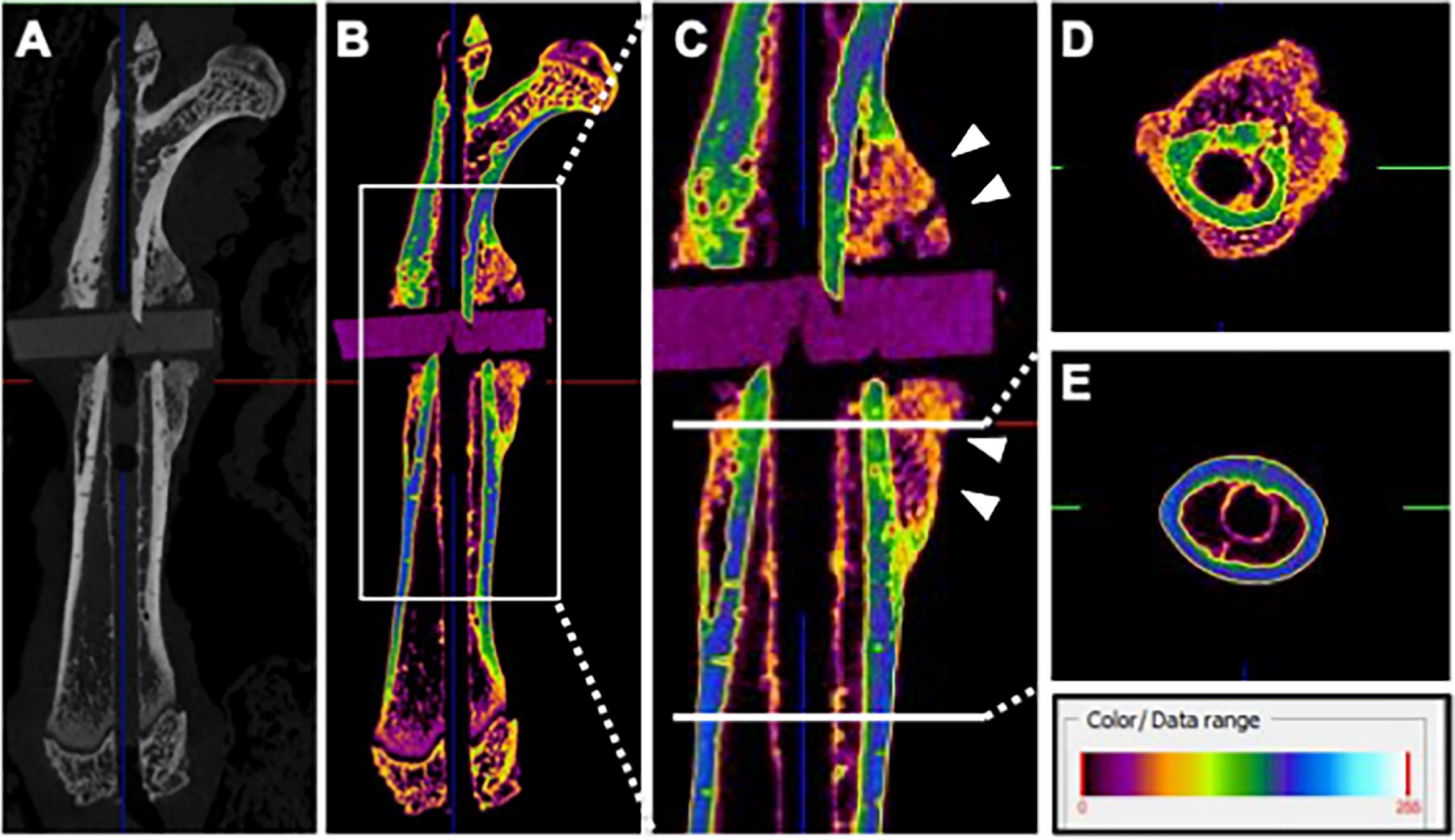
Micro-CT (μCT) images of the femur three weeks after the first operation (the intramedullary Kirschner wire was removed to avoid metal artefact effects). a) μCT image of the whole femur (grayscale image). b) μCT image of the whole femur (pseudo-colour image). c) Callus formation around the fracture site (indicated by white arrows). d) Pseudo-colour image of the cortical bone at the fracture ends, based on Hounsfield unit (HU), showing the low HU value of cortical bone and callus formation in the surrounding tissue. e) Pseudo-colour image of the normal cortical bone.

To evaluate the fracture site in our nonunion model, we performed µCT and histological analyses three weeks after the first surgery. µCT analysis showed that the Hounsfield unit of the cortical bone at the fracture ends was lower than that of the nonoperated site (Figures 2a to 2e), suggesting atrophy of the cortical bone at the fracture ends. Additionally, callus formation around the fracture site was observed (Figures 2c and 2d), possibly promoted by the rotational mechanical instability caused by intramedullary K-wire fixation.

**Fig. 3:**
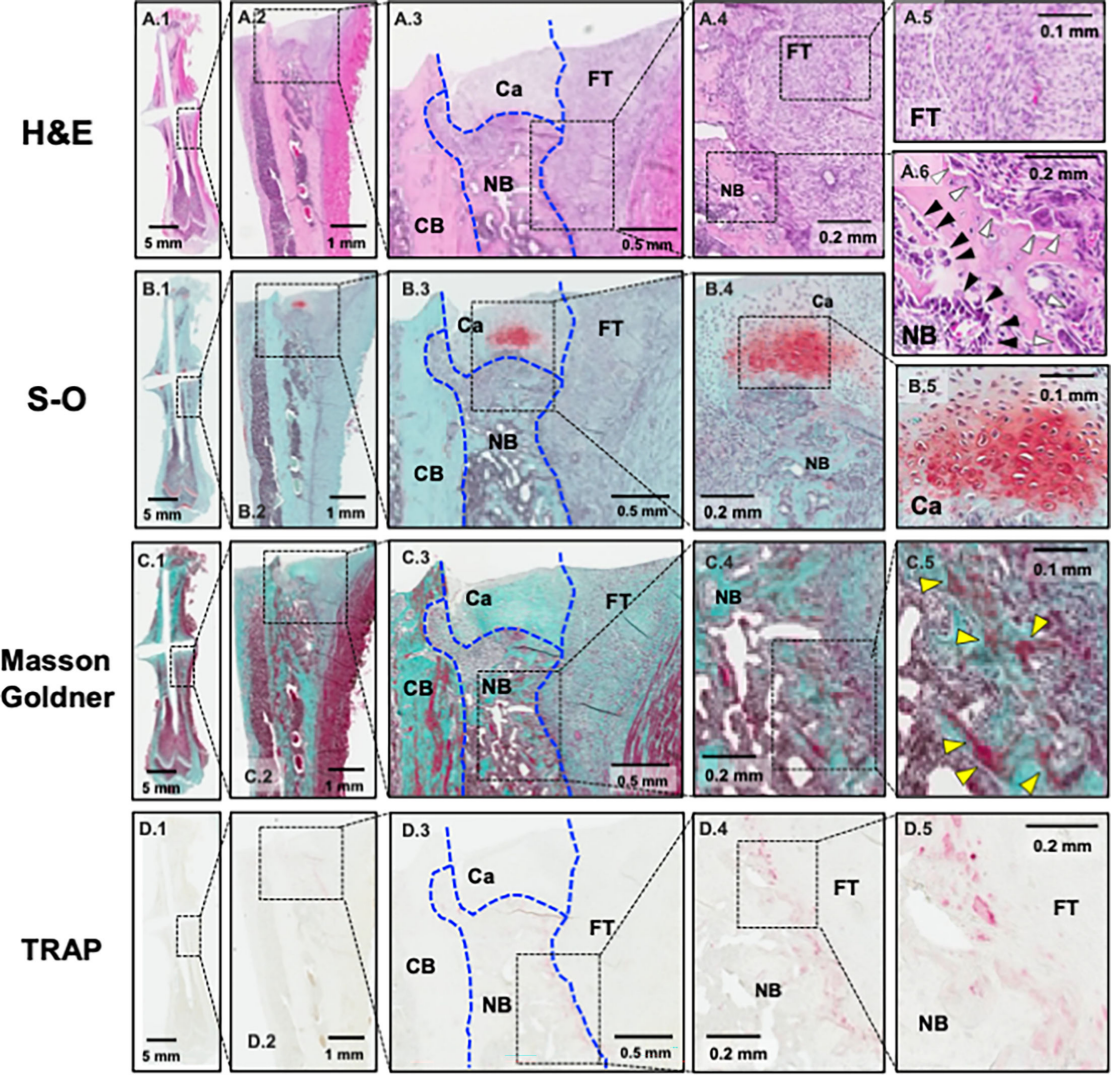
Histological images of the femur three weeks after the first operation. a1) to a6) Haematoxylin and eosin (H&E) staining. b1) to b5) Safranin-O (S-O) staining. c1) to c5) Masson-Goldner trichrome staining. d1) to d5) Tartrate-resistant acid phosphatase (TRAP) staining. a1), b1), c1), and d1) View of the whole femur. a3, b3, c3, and d3) Newly formed woven bone, cartilaginous cover, and fibrous tissue around the cortical bone at the fracture site. a5) Spindle-shaped fibroblastic cells in fibrous tissue. a6) Aligned cuboid-shaped osteoblasts (black arrows) and multinucleated osteoclasts (white arrows) around newly formed woven bone. b5) Chondrocytes in cartilage tissue. c4) and c5) Woven bone stained red by Masson-Goldner trichrome staining (yellow arrows). d4) and d5) TRAP-positive osteoclasts observed primarily on the surface of newly formed woven bone in contact with fibrous tissue. Ca, cartilaginous cover; CB, cortical bone; FT, fibrous tissue; NB, newly formed woven bone.

Histological analysis showed that the callus consisted of newly formed woven bone (hard callus) and a cartilaginous cover (soft callus). Furthermore, a large amount of fibrous scar tissue had formed around the callus. Aligned osteoblasts were observed on the newly formed woven bone, whereas TRAP-positive osteoclasts were primarily seen on the surface of the newly formed bone covered with fibrous scar tissue (Figure 3).

### Micro-CT analysis six weeks after the second surgery

**Fig. 4:**
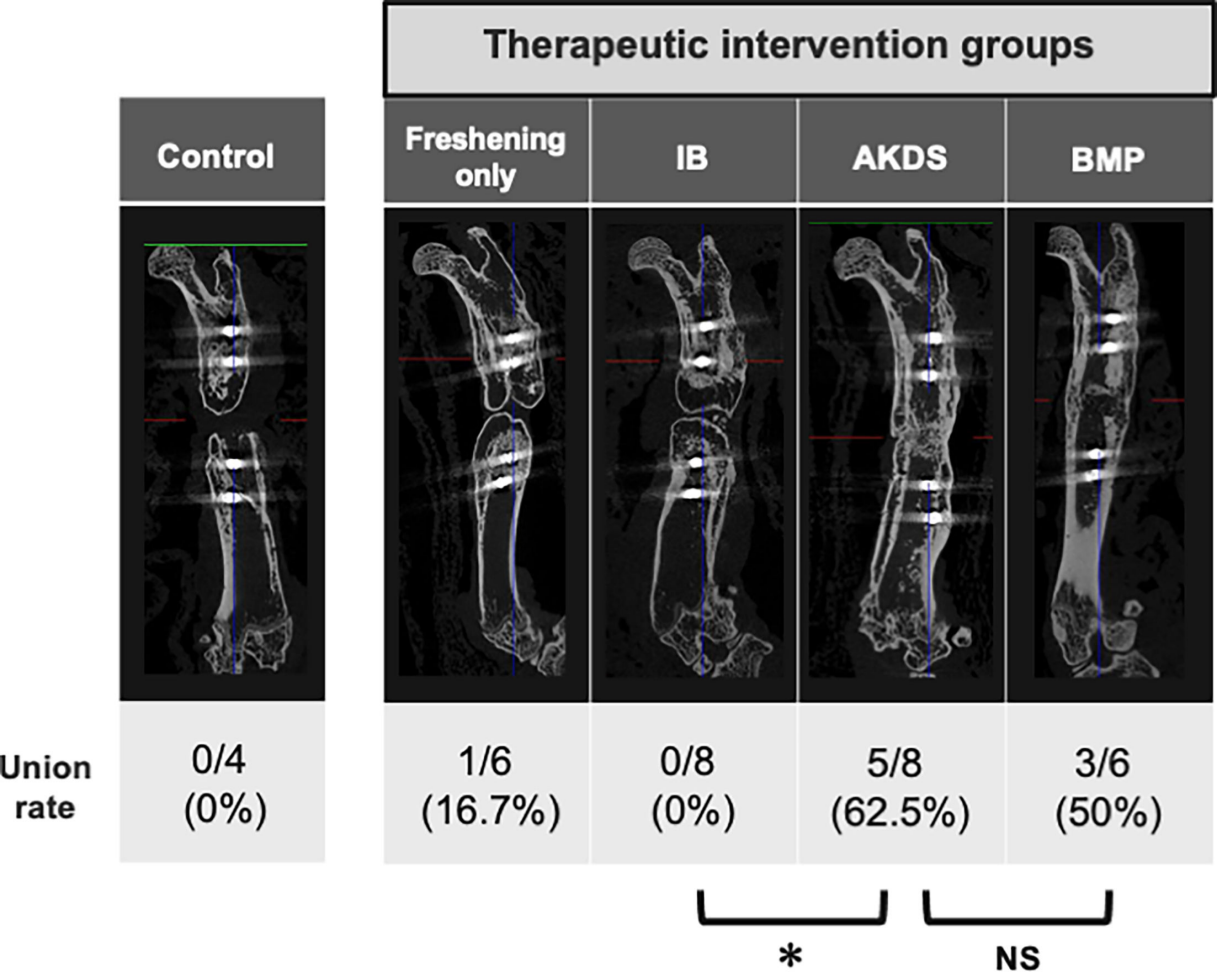
Representative micro-CT images and union rates of control and four different intervention groups. The union rate of the AKDS group (n = 5/8, 62.5%) was significantly higher than that of the iliac bone (IB) group (n = 0/8, 0%), and was comparable to that of the bone morphogenetic protein (BMP) group (n = 3/6, 50%). *p = 0.026, all p-values calculated with Fisher’s exact test. ns, not significant.

In the control group, the radiological union rate was 0%, and no healing was observed at the fracture site, validating this animal model as a novel nonunion model. The union rates in the four treatment groups (freshening, IB, AKDS, and BMP groups) were 16.7% (1/6), 0% (0/8), 62.5% (5/8), and 50.0% (3/6), respectively (Figure 4). The low union rates in the freshening and IB groups suggest that this model is a refractory nonunion model. While IB grafting alone did not achieve union, the combination of AKDS001-MS and IB grafting significantly improved the union rate (p = 0.026, Fisher’s exact test). The union rate in the AKDS group was comparable to that in the BMP group (p > 0.05, Fisher’s exact test).

**Fig. 5:**
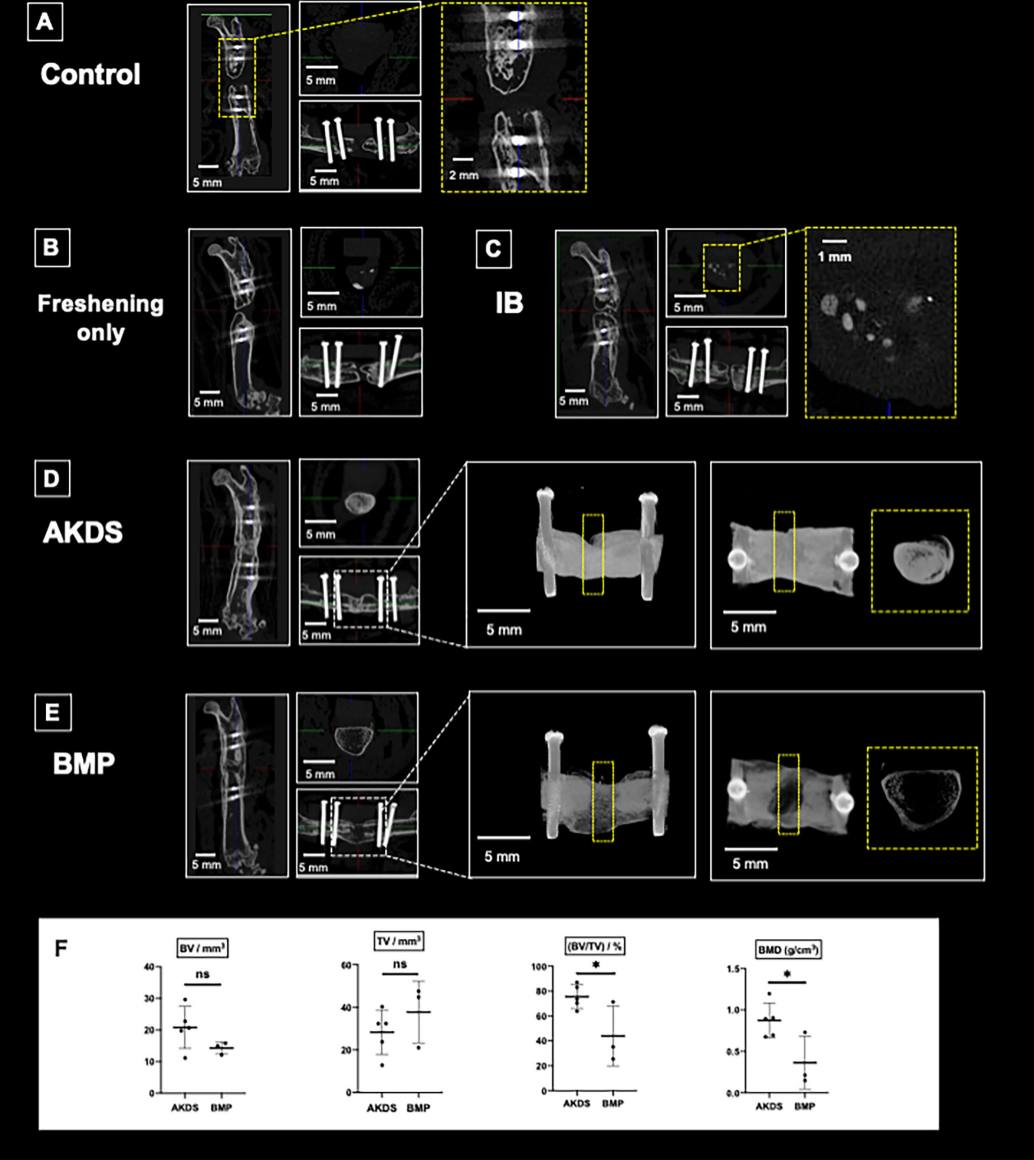
Micro-CT analyses of the defect sites in various groups. a) Control group (no grafting nor freshening). Bone union was not achieved. Little or no new bone was observed in the defect. b) Freshening only group (no grafting after freshening of fracture ends). Bone union was not achieved. c) Iliac bone (IB) grafting group. Bone union was not achieved, and some grafted bone remained in the gap. d) AKDS group (IB grafting mixed with AKDS001-MS). Bone union was achieved with dense new bone tissue. e) BMP group (bone morphogenetic protein-2 loaded collagen sponge). Bone union was achieved with large but scant shell-like new bone. f) Bone morphometric analysis of the successfully fused samples (AKDS group, n = 5; BMP group, n = 3). *p < 0.05, all p-values calculated with independent-samples *t*-test. BMD, bone mineral density; BV, bone volume; ns, not significant; TV, tissue volume.

Representative µCT images are depicted in Figure 5. Bone union was not achieved in the control, freshening only, and IB groups (Figures 5a to 5c). In the IB group, small amounts of grafted bone remained in the defect, with most of the grafted bone having disappeared (Figure 5b). In these groups, new bone formation was poor in the defect where the silicon disc had originally been interposed, suggesting that the osteogenic ability at the defect is similar to that in human atrophic nonunion. While union rates in the AKDS and BMP groups were comparable, the quality of new bone was quite different. Dense new bone filled with thick trabecular bone formed in the AKDS group (Figure 5c), in contrast to large but scant new bone that formed in the BMP group (Figure 5d). Bone morphometric analysis of the fused samples in the AKDS (five samples) and BMP (three samples) groups showed that the BV/TV ratio and BMD values in the AKDS group were significantly higher than those in the BMP group; however, BV and TV were not different between the groups (BV, p = 0.158; TV, p = 0.321; BV/TV, p = 0.035; and BMD, p = 0.032, all independent-samples *t*-test) (Figure 5e). µCT images of these samples are depicted in Supplementary Figure a.

### Histological analysis six weeks after the second surgery

**Fig. 6:**
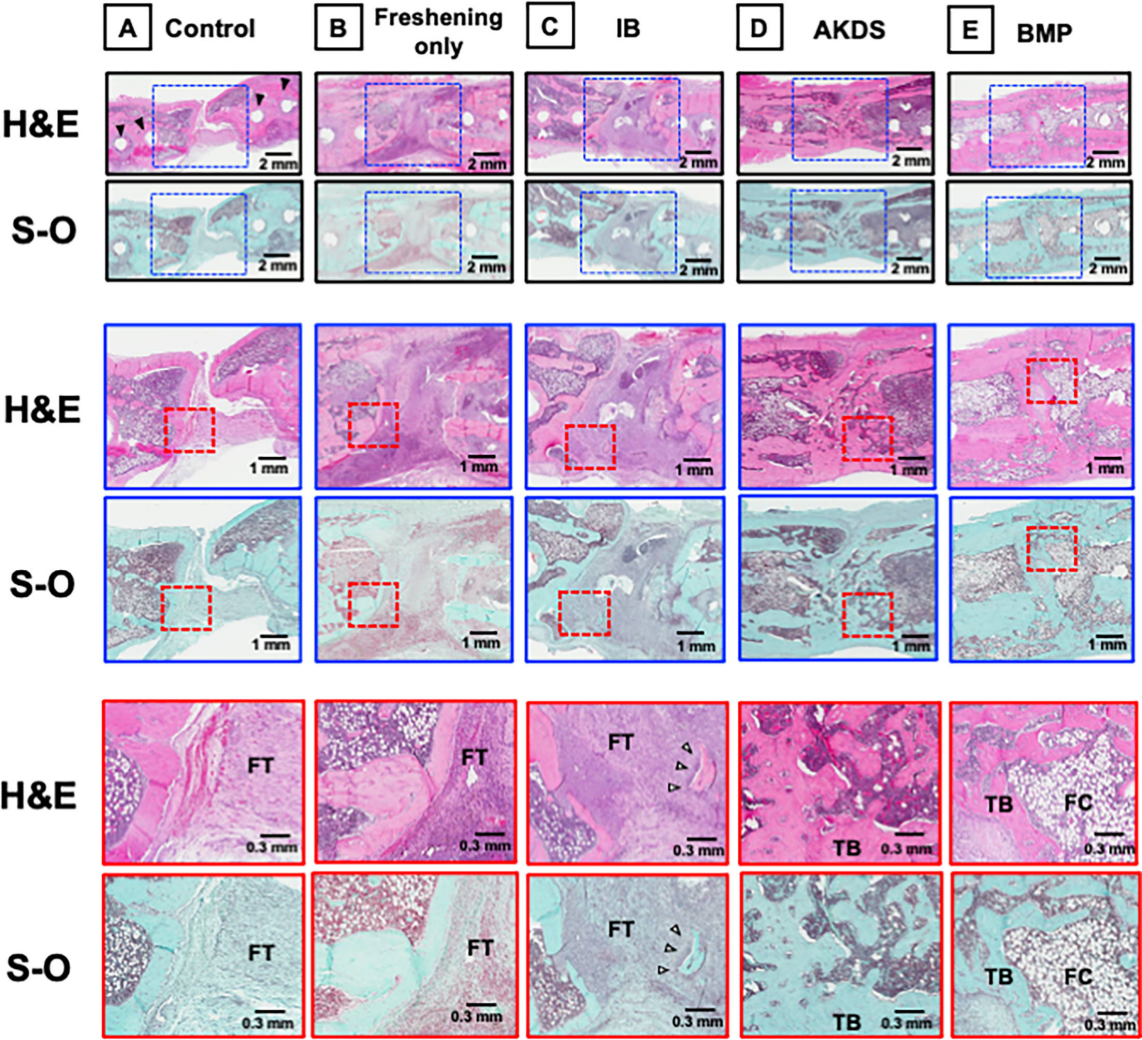
Haematoxylin and eosin (H&E) and Safranin-O (S-O)/Fast Green staining images. a) Control group (no grafting). The defect was filled with fibrous tissue (FT). Black arrows indicate screw holes. b) Freshening only group. The defect was filled with FT and minimal new bone. c) Iliac bone (IB) group. The defect was filled with FT and some residual grafted bone (white arrow). d) AKDS group (IB grafting mixed with AKDS001-MS). New bone with a dense trabecular bone (TB) structure was formed. e) Bone morphogenetic protein (BMP) group (BMP-2 loaded collagen sponge). New bone with abundant fatty cells (FC) was formed.

Representative histological images of the control, IB, AKDS, and BMP groups are depicted in Figure 6. In the control group, the defect was primarily filled with fibrous tissue (Figure 6a). Newly formed bone and cartilaginous tissue were very scarce, suggesting that the osteogenic process in the defect was atrophic. Similarly, in the freshening only group, the defect was filled with fibrous tissue and little new bone formation was observed (Figure 6b). In the IB group, the defect was mostly filled with fibrous tissue with some residual grafted bone (Figure 6c). In the AKDS group, new bone with a dense trabecular structure had formed (Figure 6d). In the BMP group, bone with scant trabecular bone and abundant fatty bone marrow was evident (Figure 6e).

## Discussion

In this study, we developed a novel rat model of long-bone nonunion using a unique two-stage surgical technique without creating a critical-sized bone defect. This model reflects the chronic condition of human nonunion by simulating a non-fresh fracture with poor osteogenic potential at the fracture ends. The 0% union rate in the control group confirmed that the bone defect in this model could not heal without therapeutic intervention. Additionally, we demonstrated the potential efficacy of AKDS001-MS as a novel therapeutic agent for bone nonunion when combined with IB grafting. AKDS001-MS significantly enhanced the union rate compared to IB grafting alone and showed superior bone regeneration quality compared to rhBMP-2 treatment.

The three-week interposition of the silicon disc at the fracture site during the first-stage surgery created biological conditions similar to those in human nonunion, such as cortical bone end atrophy, immature callus formation, and extensive fibrous scar tissue. These findings partially mirror the histological characteristics of human nonunion.^20-22^

During the second-stage surgery, the silicon disc was removed, and the fixation was converted to plate fixation to simulate rigid internal fixation used in human nonunion treatment while maintaining the 2 mm defect. Previous studies on plate fixation for fresh 2 mm bone defects reported union rates between 14% and 80%.^23-25^ However, in this nonunion model, minimal bone and cartilage tissue formation was observed in the defects, similar to atrophic bone formation, and the 2 mm bone defect did not heal in the control group. The three-week interposition of the silicon disc likely reduced the osteogenic potential at the fracture site compared to a fresh fracture. This is supported by our findings, where animals treated with IB grafting alone did not achieve bone union, and osteogenic agents (BMP-2 or EP4 agonist) were required to attain a union rate of > 50%.

Representative animal models of nonunion reported in the past include a periosteum cauterization model and a critical-sized bone defect model.^15,26^ In the periosteum cauterization model, a transverse fracture is created in a rat femur and then the periosteum of the fracture site is cauterized.^26^ This model can create atrophic changes such as resorption of cortical bone ends and fibrous tissue formation at the fracture site at eight weeks postoperatively, and the pathological findings and duration for nonunion creation are similar to our model. Although this model is simple and reproducible, the cauterization of the periosteum does not reflect clinical nonunion. The other representative animal model, the critical-sized bone defect model is simple, creating bone defects that do not heal naturally (in rats, most bone defects are ≥ 6 mm) and is as reproducible as the periosteal cauterization model.^15^ However, because the therapeutic intervention (bone graft or biomaterial graft) is performed simultaneously with the formation of the bone defect, the bone regenerative capacity of the tissue surrounding the fracture site is not compromised and does not reflect a clinical nonunion in which the environment for bone formation is harsh.

BMPs with collagen carriers have been clinically applied as alternatives to autologous bone grafting, and our nonunion model results also support the efficacy of BMP-2. The union rate in the AKDS group (n = 5/8, 62.5%) was comparable to that in the BMP group (n = 3/6, 50%). Previous studies have shown that AKDS001-MS enhances bone formation by minimizing resorption of grafted bone and promoting new bone formation.^18^ In this nonunion model, AKDS001-MS likely acted on the grafted bone, promoting bone formation even in a poor osteogenic microenvironment, similar to a pseudoarthrosis lesion. In contrast, BMPs induce bone formation by stimulating mesenchymal stem cells in the surrounding tissues to migrate and differentiate into osteoblasts,^27^ leading to bone formation from the periphery of the implanted carriers and resulting in poor bone quality inside the regenerated bone.^28^ AKDS001-MS, combined with grafted bone, may have promoted bone formation from within the defect, resulting in bone healing with a dense trabecular bone structure. The efficacy of AKDS001-MS combined with bone grafting exceeded our expectations, as AKDS001 does not possess osteoinductive activity like BMP-2. AKDS001 may have pharmacological properties that improve pathological conditions preventing bone healing in nonunion. Further investigations to elucidate the mechanisms of action of AKDS001 are ongoing.

First, the sample size was small and uneven, resulting in low statistical power. Hence, this study is at the preliminary study stage and further research with additional sample size and evaluation timeframes is currently underway. Second, this nonunion model is not strictly defined as an atrophic nonunion model, which is clinically relevant because atrophic nonunions generally do not form callus tissue.^3^ In this model, callus formation was observed after the first surgery, but biological activity at the fracture site gradually diminished during the three-week silicon disc interposition, eventually leading to atrophic-like bone formation at the defect after the second surgery. Further investigation is necessary to validate this model as an atrophic nonunion model. Third, the complexity of this model requires two precise surgical interventions, with the second surgery needing the cooperation of at least two skilled surgeons for accurate plate fixation.

In conclusion, we developed a novel animal model for long-bone nonunion using a two-stage surgical technique that reflects the clinical pathology of human nonunion. The use of AKDS001-MS in combination with bone grafting may be an effective therapeutic strategy for treating nonunion.

## Data Availability

The data that support the findings for this study are available to other researchers from the corresponding author upon reasonable request.
